# Optimization of physical energy and velocity allocation for cyclists in road cycling individual time trial using genetic algorithm

**DOI:** 10.3389/fphys.2025.1683815

**Published:** 2025-10-28

**Authors:** Xinyu Li, Benxu Zou, Xin Wang, Chaoran Liu

**Affiliations:** ^1^ Shenyang Sport University, Shenyang, China; ^2^ Hangzhou Dianzi University, Hangzhou, China

**Keywords:** road cycling individual time trials, energy and speed optimization strategy, genetic algorithm, corners, slopes

## Abstract

**Introduction:**

Effective energy management for optimizing energy and speed allocation for athletes in road cycling individual time trials is crucial due to the race’s long distances. Existing strategies often consume excessive body energy due to inadequately addressing the impact of slopes and curves.

**Methods:**

We propose an advanced energy allocation strategy using a genetic algorithm. Our research focuses on optimizing speed and energy allocation specifically in curves and on slopes given factors such as air resistance, friction, gravity and weather to maximize athletes’ energy efficiency during time trials. For curve optimization, we optimize the athletes' cornering strategies based on the parameters including road width, inner curve radius and curve angles.

**Results:**

The simulation results demonstrate that time is reduced by 9.7% on a standard 400-m track and time is reduced by 6.35% on bridge testing comparing with pre optimization strategies.

**Discussion:**

We validate the optimizing strategy based on the 2024 Paris Olympic Games road cycling individual time trial course, which demonstrates the effectiveness of the strategy. This research provides athletes with valuable guidance for optimal energy distribution.

## 1 Introduction

Cycling has emerged as a global phenomenon, revolutionizing transportation and evolving into a highly popular sport. Road cycling includes team and individual time trials. Notably, the road cycling individual time trial differs from short-distance time trials. In real road cycling individual time trials, the racecourses are typically selected to feature significant terrain variations, including curves and gradients (Wang et al., 2022), with races often spanning a considerable duration (ranging from 20 to 60 km). In such trials, the distribution of physical energy assumes a crucial role. However, existing energy allocation strategies frequently fall short in effectively accounting for corners and slopes, and inadequately consider the fluctuations in routes and environmental factors. These limitations result in suboptimal energy utilization and impede cyclists from attaining peak performance during races.

Numerous studies have adopted standardized competition plans, thereby overlooking the individual disparities (such as fitness levels, riding positions, and climatic adaptations) among different cyclists. Different cyclists may demand distinct plans in terms of fitness and tactical strategies, yet this aspect remains unaddressed in many investigations. There are numerous precedents for using mathematical models to simulate and analyze the process of road cycling. For instance, Di Prampero and Swain (Swain, 1997) studied and published a model of road cycling performance under different slope and wind conditions. The model predicts substantial time savings can be realized on hilly and windy courses by moderately increasing power on uphill or headwind segments while compensating with reduced power on downhill or tailwind segments. P. E. Di Prampero establishes a mechanical output power function for road bicycles by simulating wind and frictional resistance. Atkinson et al. (2007) verified, analyzed, and optimized the model proposed by Di Prampero and Swain. The largest time savings were observed for the hypothetical cyclist with the lowest mean power output who could vary power to the greatest extent. G. Atkinson’s findings confirm that time savings are feasible in cycling time trials if the cyclist varies power in parallel with the hill gradient and wind direction. Jeukendrup and Martin (2001) and James Martin used a model by Martin et al. to express all performance changes as variations in the 40 km time trial performance. Martin et al. (1998) derived a mathematical model of cycling power, and the values for each model parameter were determined. A bicycle-mounted power measurement system was validated by comparing it with a laboratory ergometer. Gordon (2005) introduced a model for exertion and used it to identify the distribution of power that minimizes time while constraining the cyclist’s exertion. It is demonstrated that, for a course with a climb followed by a descent, the limitations on exertion prevent the cyclist from improving performance by shifting effort towards the climb and away from the descent. Burke (2003) conducted further research on road cycling, including longitudinal acceleration to simulate and analyze road cycling. The above results in this article are attributed to the fact that the acceleration of gravity on the uphill section has a significant impact on cyclists, causing their physical exertion to increase sharply. Cyclists have to reduce their speed to reduce physical exertion. If they consume too much physical energy while climbing, it may be excessive for them to pass through the section (Wells et al., 2013). On the downhill section, due to the assistance of gravity acceleration, additional pedaling will increase their physical exertion. Therefore, maintaining a high speed on the flat section is beneficial for improving performance. At the level of event operation, a hybrid genetic algorithm was successfully used to deconstruct the player injury problem caused by the high density of NBA schedules. By reconstructing back-to-back game sequences, genetic algorithms can significantly reduce the distribution of high-intensity events, providing algorithmic support for the scientific management of professional leagues (Dawei, 2025). He et al. (2025) proposed a vision-based pose estimation method for assessing stationary cycling movements and demonstrated its high agreement with inertial sensor data and reliability across repeated tests, offering a practical tool for accessible movement assessment in settings like homes. In this study, we employ a genetic algorithm (GA), a class of bio-inspired optimization algorithms that mimic the process of natural selection and evolution. GAs are particularly suitable for complex, non-linear optimization problems such as energy allocation in cycling, as they efficiently explore large search spaces and avoid local optima through mechanisms such as selection, crossover, and mutation.

Additionally, individual time trials are susceptible to external environmental factors, including weather, terrain, and wind speed. However, the current research has not delved deeply into the impact of these environmental factors on cyclists’ performance. Some studies have relied on traditional empirical methods or simplistic mathematical models for optimizing time trial plans, lacking the support of intelligent optimization algorithms (such as genetic algorithms and machine learning algorithms). Consequently, the accuracy and applicability of these plans are restricted, precluding more detailed and intelligent optimization of competition strategies. Finally, some researches fail to integrate with actual competitions, remaining confined to partial optimization and lacking a global perspective. This renders it challenging to effectively apply the research findings in real competitions, as the unpredictability and complexity of the competition sites make it difficult for just partial optimization plans to meet global requirements.

To address these challenges, we propose a novel energy allocation strategy specifically designed for individual time trials in road cycling. This approach aims to optimize cyclists' energy distribution throughout the race, enhancing their overall performance. A genetic algorithm is employed to determine optimal energy and speed allocation, taking into account environmental factors such as weather, terrain, and individual cyclist attributes. By calculating external forces acting on the cyclists and planning their energy use, the strategy ensures feasibility under realistic conditions. The genetic algorithm iteratively minimizes race completion time, with a particular focus on optimizing performance in corners and on slopes.

## 2 Model

### 2.1 Material

The power profiles of different types of male and female cyclists were summarized respectively based on the training data of 44 female and 144 male professional cyclists (Mateo-March et al., 2022; Valenzuela et al., 2022a). Power profiles include the maximum power a cyclist can output within various time intervals, namely, 5s, 10s, 30 s, 1 min, 5 min, 10 min, 20 min, 30 min, and 60 min. These power profiles served as a comprehensive indicator of a cyclist’s capabilities across multiple aspects including sprinting ability, the ability to generate a large amount of power in a short period, aerobic and anaerobic capacities, as well as their Functional Threshold Power (FTP) value.

The experimental test section selected a real-life bridge to simulate the uphill and downhill segments that might be encountered in an individual time trial for road cycling. The bridge has an elevation of 14.8 m, a length of 1.5 km, and a gradient of 0.987%. The curved segment was modeled after a standard 400-m track, with a total length of 400 m, a curve radius of 36.5 m, and a total curve length of 231.22 m.

### 2.2 Methods

#### 2.2.1 Equation of motion in the road bicycle race

The power generated by the cyclist is not solely utilized to propel the bicycle forward but also to counteract air resistance, the frictional force between the tires and the road surface, as well as the influence of the gravitational component forces during uphill and downhill rides. When the forces are in equilibrium, the speed stabilizes, and the power output by the cyclist equals the power required to overcome the total resistive forces. In other words, the power output by the cyclist is equivalent to the power of the resistance.

**FIGURE 1:**
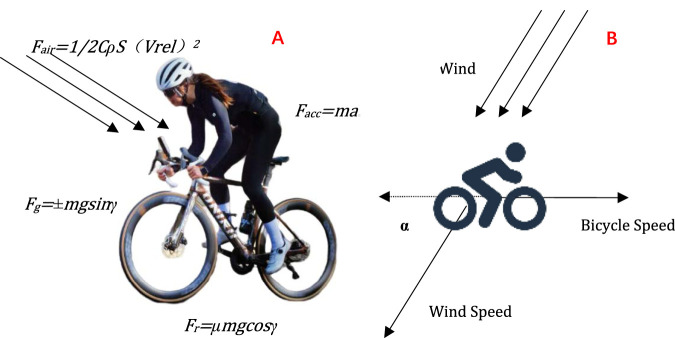
**(A)** Force analysis diagram of a road bicycle; **(B)** Influence of wind speed and wind direction on a road cyclist.

Multiple forces act simultaneously in road cycling. (Valenzuela et al., 2022b). As shown in Figure 1. *F*_*c*_ is the comprehensive external force exerted by the cyclist, while *F*_*air*_ is the air resistance during the cycling process, with the direction opposite to the direction of the ride. *F*_*g*_ is the radial force component of the rider’s gravity, which is opposite to the direction of the ride, while *F*_*r*_ is the frictional resistance, which is opposite to the direction of the ride. *F*_*acc*_ is a force that provides acceleration in the opposite direction of the riding direction. As shown in Formula 1.

$$F_{C}=F_{air}+F_{g}+F_{r}+F_{acc}$$ (1)

Furthermore, the influence of weather conditions has been taken into account in this research. Wind direction and speed are added up to the power-velocity model. In the model, the impact of air resistance on bicycle riding speed and power has been considered. Air resistance represents the reverse force exerted by the air on the moving object during riding. When investigating the influence of wind on riding, the effect of headwind is included within the air resistance. The wind resistance is defined as *F*_*air*_. The symbols used in the force analysis are listed in Table 1.

**TABLE 1:** Symbol Significance comparison table.

Symbol	Significance
*m*	Mass of people and equipment
*g*	Gravity constant
*C*	air drag coefficient (Based on weather conditions)
*S*	frontal windward area (The average value determined based on the dynamic evaluation of elite athletes' riding posture)
*V_people_*	rider’s travel speed
*V_wind_*	wind speed
*V_rel_*	relative speed of people and wind
*ρ*	gas density (Determine the atmospheric pressure based on the local latitude of Paris and the weather conditions during the competition)
*α*	angle of people and wind
*μ*	resistance coefficient of rolling friction between rubber and asphalt
$\mu_{sli}$	resistance coefficient of sliding friction between rubber and asphalt

When analyzing the influence of wind direction on riding, it is essential to comprehensively consider the direction of the wind. Not all winds are strictly downwind or headwind. The speed of the wind relative to the ground is set as Vwind. If the speed of the cyclist relative to the ground is set as Vpeople, then a vector formula can be found to calculate the relative wind speed Vrel. As shown in Formula 2.

$$V_{rel}=V_{people}-V_{wind}\cos\alpha$$ (2)

In competitions, the change of slope has a great impact on the distribution of cyclists' physical power. When analyzing cyclists' uphill and downhill riding, our model considers that the altitude and slope change continuously and smoothly. *γ* is the slope angle, m is the total mass of the cyclist and equipment, and *g* is the gravity constant. The rolling friction coefficients under dry and wet conditions are provided in Table 2.

**TABLE 2:** Rolling friction coefficient of rubber and asphalt (Yu et al., 2020).

Ground conditions	Rolling friction resistance coefficient	Average rolling friction resistance coefficient
dry	0.010–0.018	0.014
wet	0.0022–0.005	0.0036

The force required for cyclists to achieve different accelerations also varies. *F*_*acc*_ is the force that generates acceleration. Therefore, *Fc* is shown in Formula 3:

$$F_{C}=\frac{1}{2}C\rho S{\left(V_{rel}\right)}^{2}+\mu mgcos\gamma +mgsin\gamma +ma$$ (3)

#### 2.2.2 Cornering strategy

Geometrically, we consider a velodrome comprising straights, circular arcs, and connecting transition curves. As Fitzgerald (Fitzgerald et al., 2021) indicated, the inclusion of these elements, which has been commonly overlooked in previous studies, presents a mathematical challenge. However, it enhances the empirical adequacy of the model.

Cyclists do not blindly accelerate to minimize race time. Instead, they need to decelerate on sharp turns and pass at a safe speed *V* to ensure their safety. For the calculation of the safe speed during turning, we assume that the cyclists approximately execute the circular motion. For safety reasons, setting a maximum speed limit is of utmost importance. According to the model, the turning speed and the static friction coefficient between the tire and the ground are related to the turning radius. The specific formula is shown in Formula 4.

$$F=m\frac{V^{2}}{r}$$ (4)

It can be seen that the turning speed of players is closely related to the quality of tires and the choice of turning radius. Then, to make the rider complete the race in a shorter time, we optimized the turning process and obtained the optimal route for the rider to turn. To let the contestants turn at the fastest speed, they should choose a correct turning radius, which should be the radius of the largest circle where the two roads cross. This also minimally limits the maximum speed of the contestant in turning.

**FIGURE 2:**
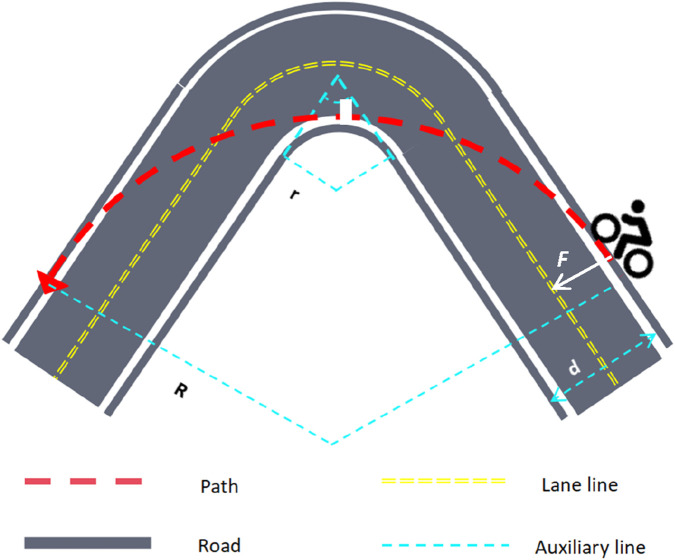
The optimal path for cornering.

The turning speed of the cyclist is closely associated with the tire quality and the choice of turning radius. Subsequently, to enable the cyclists to complete the race in a shorter time, we optimized the turning process and determined the optimal turning route. To allow the cyclists to turn at the fastest speed, they should select the correct turning radius, which is the radius of the largest circle where the two roads intersect. This choice also minimally restricts the maximum turning speed of the cyclists. The optimal turning path is illustrated in Figure 2, where the cyclist follows a trajectory that maximizes the turning radius.

According to the figure, the maximum turning radius *R* of the cyclist is shown in Formula 5.

$$R=r+\frac{d}{1-\sin\frac{\theta }{2}}$$ (5)

In the model, when a cyclist makes a turn, we define the moment of entering and leaving the corner as the starting and ending points of the arc, respectively. At this time, the path of the cyclist is tangent to the outer boundary of the road. During the intermediate stage of the turn, the vertex of the inner boundary of the road is tangent to the arc, thereby achieving the optimal turning route. In the calculation process, to obtain the optimal radius, it is necessary to know the width d of the road, the intersection angle θ of the two roads, and the radius r of the inner bend of the road. As shown in Formula 6.

$$\mu_{sli}mg=m\frac{{V_{mb}}^{2}}{R}$$ (6)

$\mu_{sli}$ is the coefficient of sliding friction between rubber and asphalt. It is 0.9 on dry roads and 0.4 on slippery roads. Therefore, the expression of *V*_*mb*_ is shown in Formula 7.

$$V_{mb}=\sqrt{\mu_{sli}gr+\frac{\mu_{sli}gd}{1-\sin\frac{\theta }{2}}}$$ (7)

#### 2.2.3 Cyclists’ physical fitness

In the task of cyclist’s physical fitness allocation, it is essential to consider the constraints on each variable. *P* is the power level applied by the cyclist at time *t*. Cyclists are incapable of generating unlimited power for movement; thus, there must exist a maximum value to constrain their speed (Wu, 2013). However, in reality, the limited physical capacity of cyclists is insufficient to sustain the entire competition at maximum power (Shoman and Imine, 2020). While limiting the total technique of the cyclists, the speed of an elite cyclist in the same event should be used for limitation.

Recovery power is the power at which an athlete can perform work indefinitely based on aerobic metabolism without considering muscle fatigue, provided that P is a certain power value higher than *P*_*recover*_. When P is less than *P*_*recover*_, *P*_*recover*_ is a power that a cyclist can maintain for an extended period; that is, pedaling at or below this limit can, theoretically, be continued indefinitely. The average and maximum speeds of elite male and female cyclists are summarized in Table 3.

**TABLE 3:** Speed of elite road bicycle individual time trial cyclists.

Gender	Average velocity (m/s)	Maximum speed (m/s)
male	15.00	20.83
female	13.61	18.06

#### 2.2.4 Genetic algorithm (GA)

Our mode is specifically applicable to cyclists who employ high-power cycling early in time trials, because maintaining the cyclist’s output power can effectively alleviate fatigue caused by the variability of the terrain during actual competitions (Atkinson et al., 2007). There are distinct riding plans for different terrains. How to design a reasonable power management strategy? On the premise of ensuring that the total energy consumption of the cyclist does not exceed the limit, the final performance evaluation index is the total time taken from the starting point to the key point. Considering the influences of various factors such as different terrains, wind forces, and the constant total energy of the cyclists on the riding strategy, the genetic algorithm (GA) is employed to minimize the total riding time in real road timing competitions. During the optimization process, all relevant constraints are taken into account.

The sections can be divided according to a certain distance for the actual competitions. For example, an almost flat road with some low slopes can be regarded as a flat road with a slope angle of 0. The race section with a small curve can be considered as a straight line. For the hillside terrain, the connection between the top and bottom of the slope is treated as a slope. After the above processing of the competition section, the route in a competition can be divided into sections. Then, the solution of changing the power (speed) according to the competition distance is adopted, and the cyclists use constant power in the same section. From the analysis of the motion equation, it can be concluded that there is a constant relationship between cyclists' output power and speed, which is relevant to internal and external factors such as slope, length of divided distances, and bending angles. The determined speed can be obtained by changing the power. Therefore, we can do discretization processing for each segment of speed and power. We can discretize the speed and power of each segment to obtain the P_i_ and v_i_ corresponding to each segment.

**FIGURE 3:**
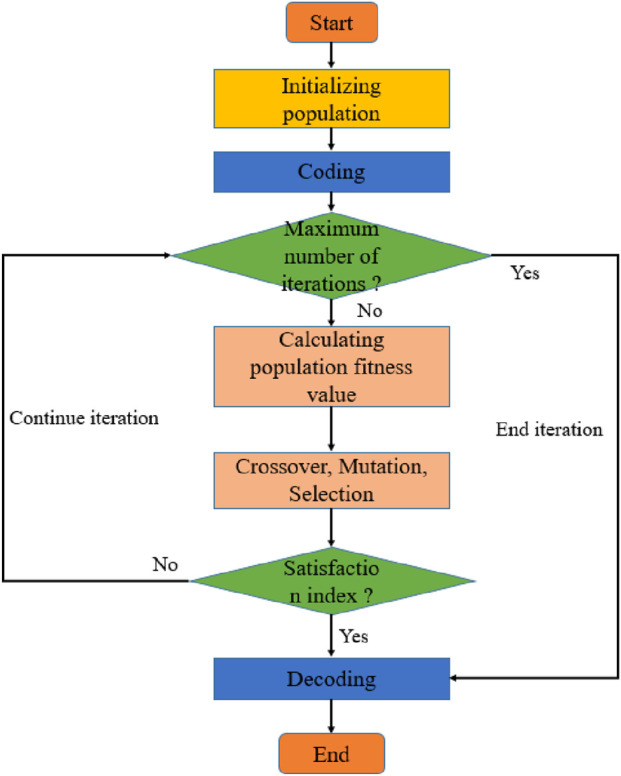
Flow Figure of genetic algorithm.

Several initial populations encoded by a certain length are generated randomly. Each individual was evaluated by the fitness function.In the implementation of the genetic algorithm, a real-valued encoding scheme was employed. The term “Encoding” refers to the representation of a potential solution to the optimization problem—namely, a vector containing the velocity values for all road segments, V = [v_1_, v_2_, …, v_n_]—as an individual (or chromosome) within the population. Since velocity is a continuous variable, real-valued encoding is more direct and efficient than binary encoding for this problem. The term “Decoding” refers to the process of translating this individual (the velocity vector) into the objective and constraint values. This is done by substituting the velocity values into the fitness function (Equation 8) and the system of constraint equations (e.g., Equations 9, 10) to calculate the total race time and energy consumption, thereby evaluating the individual’s fitness. Each individual thus directly represents a complete velocity allocation strategy for the entire course. Individuals with high fitness values were selected to participate in the genetic operation, while those with low fitness were eliminated. A new generation of population is formed by the collection of genetically manipulated individuals. The real-valued linear recombination method was used in the genetic process. At the same time, the real-valued mutation was used in the mutation process, in which the population size is set to 500, the maximum number of iterations is set to 200, the mutation probability is set to 0.2, and the crossover probability is set to 0.8. Until the stop criteria are met, the process is shown in Figure 3. The fitness function is shown in Formula 8.

$$f\left(x\right)=\frac{1}{\sum_{i=1}^{nVar}x\left(i\right)}$$ (8)

The time required for each segment can be determined, and then time can be summed up to obtain the total time required for the cyclists’ movement process. The total riding time is optimized by using the established model. The constraint conditions of the optimization model are shown in (Equation 9), and the objective function is shown in (Equation 10).

$$V_{1}=x\left(count\right)$$

$$V_{rel}=V_{1}-V_{wind}\times \cos\left(\left|D_{wind}-D_{seg}\left(i\right)\right|\right)$$

$$F_{air}=0.5\times C\times \rho \times S_{wind}\times {V_{rel}}^{2}$$

$$F_{g}=miu\_roll\times m\times \cos\left(\gamma \left(i\right)\right)$$

$$F_{r}=m\times g\times \sin\left(\gamma \left(i\right)\right)$$

$$F_{c}=F\_air+F\_g+F\_r$$

$$V_{rec}=\frac{P_{recover}}{F_{c}}$$

$$c\left(count\right)=V_{rec}-V_{1}$$

$$\begin{array}{ll} & \;W\left(i\right)=F_{c}\times s\left(i\right)-P_{recover}\times \frac{s\left(i\right)}{V_{1}}\\ & \;cep=W_{all}-\sum W \end{array}$$ (9)

The flow of the genetic algorithm used in this study is depicted in Figure 3.

The influence of gravity and friction forces on cyclists within a specific road section is fixed, yet different speeds will have an effect on the wind resistance endured by the cyclists. Consequently, the higher the speed is, the greater the power required to sustain it. Simultaneously, more energy will be consumed. In contrast, the performance in the descending section is not dependent on the power output. Otherwise, it will increase energy consumption. If the cyclist does not rotate the crank to complete the descent at the same speed, the recovery speed prior to the subsequent climb can be improved, which may be beneficial for the race results (Atkinson et al., 2007). Therefore, it is assumed that at the end of the competition, the total amount of the cyclist’s allocable physical capacity is a fixed value, and *W* is just 0. At this time, the *W* expression is shown in Formula 10.

$$W=\sum_{i=1}^{n}\left(F_{ci}s_{i}-P_{recover}t_{i}\right)$$ (10)

When $\sum_{i=1}^{n}t_{i}$ reaches the minimum value, that is, when the shortest time of the entire competition is achieved, it represents the best performance of the cyclists. At this point, the power P of each part is the ideal power for the cyclists when passing through each stage. However, this speed needs to be constrained in the calculation. Therefore, the upper limit of the speed on the uphill and horizontal sections is the maximum speed of elite cyclists when passing through this stage, and the lower limit is determined by the speed corresponding to their current recovery power.

The genetic algorithm selects all individuals in the population as the object, randomizes the values of all individuals under specific conditions, and efficiently searches for the required results. During the genetic process, the genetic operations of selection, crossover, and variation are employed. In the application of the genetic algorithm to cycling competitions, the velocity of cyclists in different road sections is regarded as the individual population. For instance, we use n types of randomization techniques to guide the generation of the initial velocity allocation strategy for competition venues with different road sections, and then perform binary coding to imitate chromosomes in nature. Subsequently, the initial population with a population number of N is generated. Fitness reflects the competitiveness of individuals. Individuals with higher fitness have a greater probability of survival in the selection process. The goal of the genetic algorithm is to find the power allocation strategy corresponding to the minimum t. Therefore, the smaller the individual t is, the smaller the corresponding fitness will be as well. In the inheritance process of the genetic algorithm, the real-value linear recombination method is adopted. At the same time, the variation process uses real-value variation.

## 3 Results and discussion

### 3.1 Validation of the model curve

**FIGURE 4:**
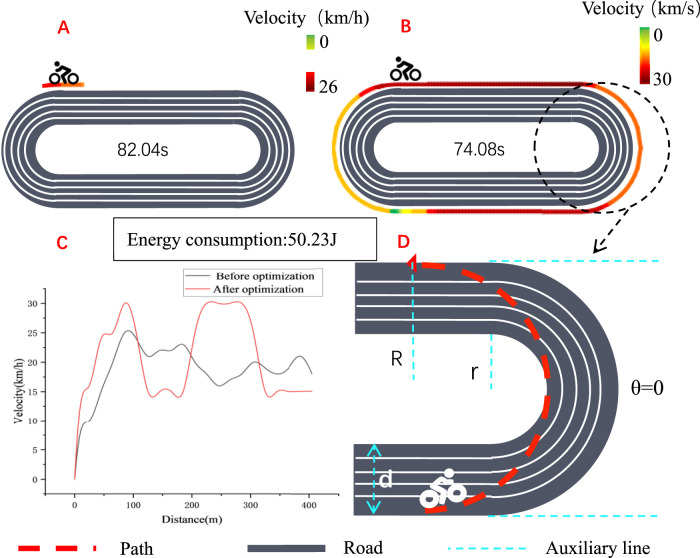
**(A)** Speed distribution on the track before optimization; **(B)** Speed distribution on the track after optimization; **(C)** Speed comparison before and after optimization; **(D)** Schematic diagram of cornering calculations optimization

Based on the tests implemented by the experimenters on a standard 400-m track, the results are shown in Figure 4. It took a total of 82.04 s in the pre-optimization test. After the model was optimized, with the same energy consumption, the test result was 74.08 s, indicating a performance improvement of 9.7%.

### 3.2 Validation of the model slope

**FIGURE 5:**
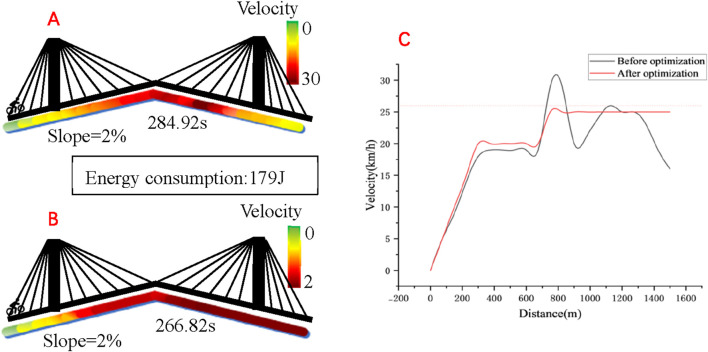
**(A)** Track speed distribution before optimization; **(B)** Track speed distribution after optimization; **(C)** Speed comparison before and after optimization.

According to the tests conducted by the experimenters over a distance of 1500 m, the results are depicted in Figure 5. It took a total of 284.92 s in the pre-optimization test. After the model was optimized, with the same energy consumption, the test result was 266.82 s, indicating an improvement in performance of 6.35%.

### 3.3 The individual time trial for road cycling at the Paris 2024 Olympic game

**FIGURE 6:**
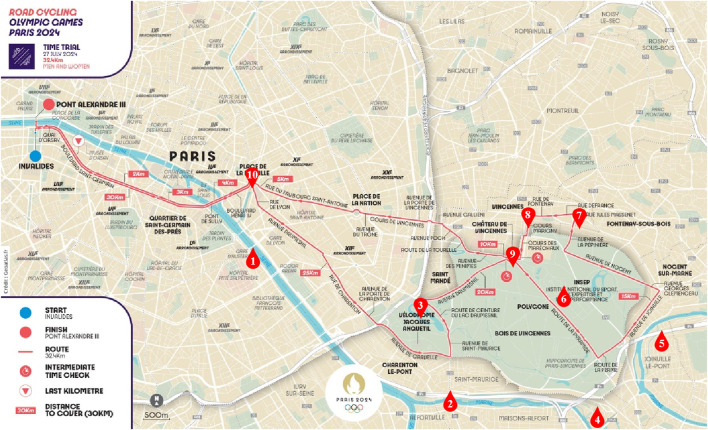
The course for the Individual Time Trial in Road Cycling at the Paris 2024 Olympic Games.

**FIGURE 7:**
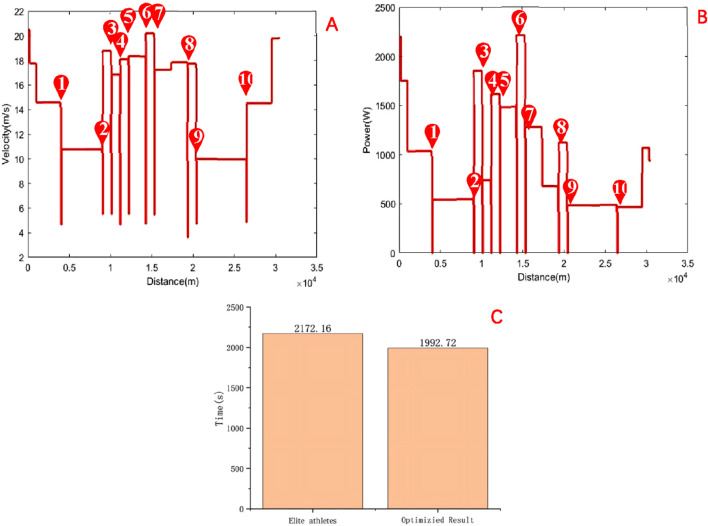
**(A)** Speed distribution after optimization; **(B)** Power distribution after optimization; **(C)** Comparison of race time after optimization with the winning time in the Paris Olympic Games road cycling individual time trial.

The competition map is shown in Figure 6. After optimization, the research findings indicate that the performance of male road cycling athletes is 33 min and 12.72 s, representing a 6.62% improvement over the winning time of 36 min and 12.16 s achieved by the men’s champion at the 2024 Paris Olympics. The result is shown in Figure 7. The improvement not only demonstrates the potential of the optimization strategy in improving athlete performance but also emphasizes the significance of effective power distribution and speed management. However, it is important to note that the actual performance of cyclists in competitive settings may be influenced by various factors, such as weather conditions, road surface, and psychological state, which may differ significantly from the simulated conditions on which the model is based. Therefore, to ensure the model’s accuracy and applicability, further research will require validation of the simulation results through empirical measurements and field testing. This approach will aid in refining the model to better align with real-world competitive environments, ultimately providing more scientific guidance for cyclists’ training and racing strategies in future competitions.

## 4 Discussion

This article synthesizes a comprehensive dataset on the competition and training performances of elite male and female cyclists, systematically analyzing the optimal power output and speed across diverse slopes, wind directions, and wind intensities. Based on this in-depth analysis, an optimal physical fitness allocation strategy for elite road cyclists is proposed to enhance their performance during competitions. The constructed model was rigorously simulated to evaluate its effectiveness, and multiple tests have confirmed that using a genetic algorithm for physical energy allocation can improve their performance under identical track conditions. However, limitations in venue availability and equipment have precluded the full replication of real competition environments, and further validation is urgently needed to assess the impact on cyclist performance in official events.

The above results indicate that the output power and speed of cyclists reach their maximum values on flat slopes. The finding that significant time savings can be achieved on hilly and windy courses by increasing power on uphill or headwind segments while compensating with reduced power on downhill or tailwind segments is consistent with the findings of Di Prampero et al. (1979).

There is a wide range of factors that can influence individual time trial performance. While some factors play a role in all racing conditions, others are only effective in certain circumstances. In courses with a lower proportion of technical sections, the simulation results suggest that road conditions do not significantly affect the final performance time (Zignoli, 2021). The power delivery ability is limited by the cornering angle, indicating that power output cannot be achieved during cornering. This condition has significant implications on the way of cylist’s cornering trajectories. For one thing, the roll angles are promptly restored after a corner to enable power output production. For another, corners may provide a key opportunity to recover physical fitness. The simulation results presented in Figure 5 indicate that flat and downhill technical sections demand significantly distinct average power output values because of the role of gravity in reaccelerating the system following a downhill corner.

The article still leaves some external factors unaccounted for. A cyclist cannot always adhere strictly to a strategy that approaches the physical capacity limit. Moreover, physiological measurements can be conducted during the actual trial, which can promptly reflect the changes in the optimal strategy, as the actual physiological state during the trial always varies from the state estimated by the model based on previous calibration tests (Beaumont et al., 2023). Additionally, for the speed changes of cyclists during the competition, such as the transition from low speed to high speed, additional physical energy is required to accelerate the entire system, thereby increasing the overall physical energy consumption. This process cannot be implemented through programming in this article, and the model still needs to be further optimized in the future.

## 5 Conclusion

When the cyclist is on the downhill section, generating a higher power is not beneficial for his subsequent physical recovery and can readily cause excessive physical exertion and potential dangers. Maintaining a high power output on the flat or uphill sections represents an effective physical distribution strategy to enhance his performance in the competition. For individual road bicycle races, genetic algorithms can be employed for optimization analysis to successfully develop the optimal physical distribution plan. Since genetic algorithms possess strong search capabilities through the utilization of mutation mechanisms, they can prevent getting trapped in local optima and ignoring the global situation.

## Data Availability

The original contributions presented in the study are included in the article/supplementary material, further inquiries can be directed to the corresponding authors.
